# Cyclo­hexa-2,5-diene-1,4-dione–1,2,4,5-tetra­fluoro-3,6-diiodo­benzene (1/1)

**DOI:** 10.1107/S1600536812015930

**Published:** 2012-04-18

**Authors:** Peng Liu, Chuansheng Ruan, Tiesheng Li, Baoming Ji

**Affiliations:** a Department of Chemistry, Zhengzhou University, Zhengzhou 450052, People’s Republic of China; b College of Chemistry and Chemical Engineering, Luoyang Normal University, Luoyang 471022, People’s Republic of China

## Abstract

The asymmetric unit of the title co-crystal adduct, C_6_H_4_O_2_·C_6_F_4_I_2_, comprises a half-mol­ecule each of cyclo­hexa-2,5-diene-1,4-dione and 1,2,4,5-tetra­fluoro-3,6-diiodo­benzene. The C_6_F_4_I_2_ mol­ecule is almost planar (r.m.s. deviation = 0.0062 Å). In the crystal, the components are connected through O⋯I halogen bonds [3.017 (11) Å], leading to the formation of wavelike chains along the *a* axis. The crystal packing also features C—H⋯F inter­actions.

## Related literature

For related studies on co-crystal formation, see: Bhogala & Nangia (2008) ▶; Ji *et al.* (2011 ▶); Arman *et al.* (2010 ▶); Cardillo *et al.* (2000 ▶). For background to halogen bonding, see: Metrangolo *et al.* (2008 ▶).
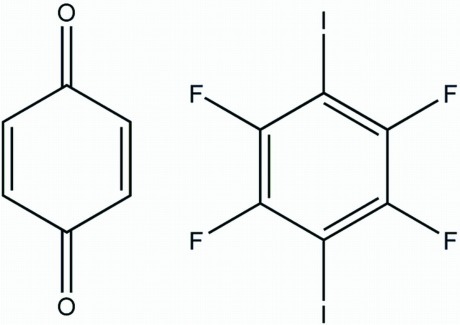


## Experimental

### 

#### Crystal data

C_6_H_4_O_2_·C_6_F_4_I_2_
*M* *_r_* = 509.95
Triclinic, 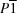

*a* = 5.778 (3) Å
*b* = 6.354 (3) Å
*c* = 10.013 (5) Å
α = 102.295 (5)°
β = 93.861 (5)°
γ = 97.781 (5)°
*V* = 354.1 (3) Å^3^
*Z* = 1
Mo *K*α radiation
μ = 4.48 mm^−1^
*T* = 296 K
0.43 × 0.30 × 0.26 mm

#### Data collection

Bruker APEXII CCD diffractometer
Absorption correction: multi-scan (*SADABS*; Sheldrick, 1996 ▶) *T*_min_ = 0.249, *T*_max_ = 0.389
2585 measured reflections
1291 independent reflections
1096 reflections with *I* > 2σ(*I*)
*R*_int_ = 0.020

#### Refinement

*R*[*F* ^2^ > 2σ(*F* ^2^)] = 0.033
*wR*(*F* ^2^) = 0.087
*S* = 1.07
1291 reflections
91 parameters
H-atom parameters constrained
Δρ_max_ = 1.34 e Å^−3^
Δρ_min_ = −0.76 e Å^−3^

### 

Data collection: *APEX2* (Bruker, 2004 ▶); cell refinement: *SAINT* (Bruker, 2004 ▶); data reduction: *SAINT*; program(s) used to solve structure: *SHELXS97* (Sheldrick, 2008 ▶); program(s) used to refine structure: *SHELXL97* (Sheldrick, 2008 ▶); molecular graphics: *SHELXTL* (Sheldrick, 2008 ▶) and *DIAMOND* (Brandenburg, 2006 ▶); software used to prepare material for publication: *SHELXTL* and *PLATON* (Spek, 2009 ▶).

## Supplementary Material

Crystal structure: contains datablock(s) global, I. DOI: 10.1107/S1600536812015930/ds2186sup1.cif

Structure factors: contains datablock(s) I. DOI: 10.1107/S1600536812015930/ds2186Isup2.hkl

Supplementary material file. DOI: 10.1107/S1600536812015930/ds2186Isup3.cml

Additional supplementary materials:  crystallographic information; 3D view; checkCIF report

## Figures and Tables

**Table 1:** Hydrogen-bond geometry (Å, °)

*D*—H⋯*A*	*D*—H	H⋯*A*	*D*⋯*A*	*D*—H⋯*A*
C6—H6⋯F1^i^	0.93	2.64	3.562	171

Symmetry code: (i) 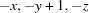.

**Fig. 1.**
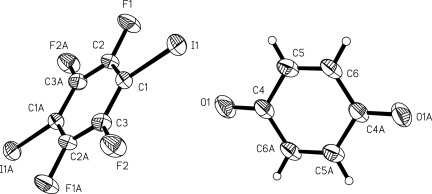
View of the title molecular structure with atom numbering scheme and 30% probability displacement ellipsoids for non-hydrogen atoms.

**Fig. 2.**
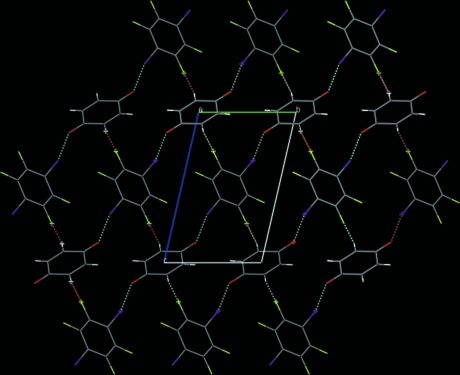
A view in projection down the b axis showing the unit-cell contents. The O—I and C—H···F are shown as bule and purple dashed lines.

## supplementary crystallographic information

### Comment

The title co-crystal is part of a study on the halogen bond, which is a powerful intermolecular interaction we and others have used extensively to produce a variety of structures involving perfuorinated compounds (Ji *et al.* 2011; Arman *et al.* 2010; Cardillo *et al.* 2000), usually very diffcult to crystallize.

In the crystal structure, 1,2,4,5-tetrafluoro-3,6-diiodobenzene molecule is flat with the *r.m.s.* deviation of the 12 constituent atoms being 0.0062 Å (Fig. 1). It is noted that the cyclohexa-2,5-diene-1,4-dione molecule acts as a bidentate donor towards 1,2,4,5-tetrafluoro-3,6-diiodobenzene molecule, giving rise to chains extended throughout the whole crystal, in which the bond length of O···I halogen bond is 3.017 Å, as observed in the previous reports (Metrangolo *et al.* 2008; Ji *et al.* 2011).

In addition, the molecules are further stabilized in the crystal packing *via* a combination of C—H···F contacts (Table. 1), as shown in Fig. 2.

### Experimental

The starting materials were commercial obtained from Aldrich. The 1:1 adduct was obtained by dissolving in chloroform, at room temperature and in a vial, equimolecular amounts of cyclohexa-2,5-diene-1,4-dione and 1,2,4,5-tetrafluoro-3,6-diiodobenzene. The open vial was closed in a cylindrical bottle containing vaseline oil. Volatile solvents were allowed to diffuse at room temperature and, after one day, the yellow block crystals were obtained.

### Refinement

All H atoms were positioned geometrically and treated as riding, with C—H bond lengths constrained to 0.93 Å (aromatic CH), and with *U*ĩso~(H) = 1.2Ueq(C).

### Crystal data

**Table:**

C_6_H_4_O_2_·C_6_F_4_I_2_	*Z* = 1
*M**_r_* = 509.95	*F*(000) = 234
Triclinic, *P*1	*D*_x_ = 2.391 Mg m^−^^3^
Hall symbol: -P 1	Mo *K*α radiation, λ = 0.71073 Å
*a* = 5.778 (3) Å	Cell parameters from 1573 reflections
*b* = 6.354 (3) Å	θ = 3.3–25.5°
*c* = 10.013 (5) Å	µ = 4.48 mm^−^^1^
α = 102.295 (5)°	*T* = 296 K
β = 93.861 (5)°	Block, yellow
γ = 97.781 (5)°	0.43 × 0.30 × 0.26 mm
*V* = 354.1 (3) Å^3^	

### Data collection

**Table:**

Bruker APEXII CCD diffractometer	1291 independent reflections
Radiation source: fine-focus sealed tube	1096 reflections with *I* > 2σ(*I*)
Graphite monochromator	*R*_int_ = 0.020
phi and ω scans	θ_max_ = 25.5°, θ_min_ = 3.3°
Absorption correction: multi-scan (*SADABS*; Sheldrick, 1996)	*h* = −6→6
*T*_min_ = 0.249, *T*_max_ = 0.389	*k* = −7→7
2585 measured reflections	*l* = −12→12

### Refinement

**Table:**

Refinement on *F*^2^	Primary atom site location: structure-invariant direct methods
Least-squares matrix: full	Secondary atom site location: difference Fourier map
*R*[*F*^2^ > 2σ(*F*^2^)] = 0.033	Hydrogen site location: inferred from neighbouring sites
*wR*(*F*^2^) = 0.087	H-atom parameters constrained
*S* = 1.07	*w* = 1/[σ^2^(*F*_o_^2^) + (0.0571*P*)^2^] where *P* = (*F*_o_^2^ + 2*F*_c_^2^)/3
1291 reflections	(Δ/σ)_max_ < 0.001
91 parameters	Δρ_max_ = 1.34 e Å^−^^3^
0 restraints	Δρ_min_ = −0.76 e Å^−^^3^

### Special details

**Table:**

Geometry. All e.s.d.'s (except the e.s.d. in the dihedral angle between two l.s. planes) are estimated using the full covariance matrix. The cell e.s.d.'s are taken into account individually in the estimation of e.s.d.'s in distances, angles and torsion angles; correlations between e.s.d.'s in cell parameters are only used when they are defined by crystal symmetry. An approximate (isotropic) treatment of cell e.s.d.'s is used for estimating e.s.d.'s involving l.s. planes.
Refinement. Refinement of *F*^2^ against ALL reflections. The weighted *R*-factor *wR* and goodness of fit *S* are based on *F*^2^, conventional *R*-factors *R* are based on *F*, with *F* set to zero for negative *F*^2^. The threshold expression of *F*^2^ > σ(*F*^2^) is used only for calculating *R*-factors(gt) *etc*. and is not relevant to the choice of reflections for refinement. *R*-factors based on *F*^2^ are statistically about twice as large as those based on *F*, and *R*- factors based on ALL data will be even larger.

### Fractional atomic coordinates and isotropic or equivalent isotropic displacement parameters (Å^2^)

**Table:**

	*x*	*y*	*z*	*U*_iso_*/*U*_eq_	
C1	0.3224 (8)	0.5732 (8)	0.4260 (5)	0.0453 (11)	
C2	0.3930 (9)	0.3726 (8)	0.3786 (5)	0.0466 (11)	
C3	0.4339 (9)	0.6981 (8)	0.5494 (5)	0.0501 (12)	
C4	0.5734 (10)	0.8493 (9)	0.0745 (6)	0.0593 (14)	
C5	0.3440 (10)	0.8029 (10)	−0.0092 (6)	0.0652 (15)	
H5	0.2464	0.6719	−0.0141	0.078*	
C6	0.2763 (10)	0.9415 (10)	−0.0761 (6)	0.0612 (14)	
H6	0.1296	0.9086	−0.1263	0.073*	
F1	0.2901 (6)	0.2428 (6)	0.2593 (3)	0.0702 (9)	
F2	0.3718 (6)	0.8922 (5)	0.6021 (4)	0.0712 (9)	
I1	0.05048 (6)	0.67187 (6)	0.31973 (4)	0.06065 (19)	
O1	0.6309 (10)	0.7170 (8)	0.1376 (5)	0.0898 (15)	

### Atomic displacement parameters (Å^2^)

**Table:**

	*U*^11^	*U*^22^	*U*^33^	*U*^12^	*U*^13^	*U*^23^
C1	0.045 (3)	0.048 (3)	0.043 (3)	0.0023 (19)	−0.003 (2)	0.014 (2)
C2	0.047 (3)	0.047 (3)	0.039 (3)	−0.002 (2)	−0.007 (2)	0.003 (2)
C3	0.054 (3)	0.045 (3)	0.047 (3)	0.003 (2)	0.001 (2)	0.004 (2)
C4	0.064 (3)	0.058 (3)	0.051 (3)	0.022 (3)	−0.013 (3)	0.000 (3)
C5	0.062 (3)	0.068 (4)	0.055 (3)	0.004 (3)	−0.011 (3)	0.001 (3)
C6	0.054 (3)	0.069 (4)	0.053 (3)	0.020 (3)	−0.017 (2)	−0.002 (3)
F1	0.076 (2)	0.067 (2)	0.0527 (18)	0.0096 (16)	−0.0214 (16)	−0.0097 (15)
F2	0.084 (2)	0.0544 (19)	0.068 (2)	0.0227 (16)	−0.0092 (17)	−0.0052 (16)
I1	0.0554 (3)	0.0678 (3)	0.0610 (3)	0.00885 (17)	−0.00837 (17)	0.02435 (19)
O1	0.108 (4)	0.073 (3)	0.085 (3)	0.027 (3)	−0.035 (3)	0.016 (2)

### Geometric parameters (Å, º)

**Table:**

C1—C3	1.381 (7)	C4—O1	1.221 (7)
C1—C2	1.388 (7)	C4—C6^ii^	1.483 (9)
C1—I1	2.079 (5)	C4—C5	1.478 (8)
C2—F1	1.346 (5)	C5—C6	1.298 (9)
C2—C3^i^	1.373 (8)	C5—H5	0.9300
C3—F2	1.340 (6)	C6—C4^ii^	1.483 (9)
C3—C2^i^	1.373 (8)	C6—H6	0.9300
			
C3—C1—C2	116.9 (5)	O1—C4—C6^ii^	123.3 (5)
C3—C1—I1	122.0 (4)	O1—C4—C5	119.7 (6)
C2—C1—I1	121.0 (4)	C6^ii^—C4—C5	117.0 (5)
F1—C2—C3^i^	118.8 (5)	C6—C5—C4	121.2 (6)
F1—C2—C1	119.7 (4)	C6—C5—H5	119.4
C3^i^—C2—C1	121.6 (4)	C4—C5—H5	119.4
F2—C3—C2^i^	118.5 (4)	C5—C6—C4^ii^	121.8 (5)
F2—C3—C1	120.0 (5)	C5—C6—H6	119.1
C2^i^—C3—C1	121.6 (5)	C4^ii^—C6—H6	119.1
			
C3—C1—C2—F1	179.7 (5)	C2—C1—C3—C2^i^	0.2 (8)
I1—C1—C2—F1	2.5 (7)	I1—C1—C3—C2^i^	177.4 (4)
C3—C1—C2—C3^i^	−0.2 (8)	O1—C4—C5—C6	179.1 (6)
I1—C1—C2—C3^i^	−177.4 (4)	C6^ii^—C4—C5—C6	−1.2 (10)
C2—C1—C3—F2	−179.0 (5)	C4—C5—C6—C4^ii^	1.3 (10)
I1—C1—C3—F2	−1.9 (7)		

Symmetry codes: (i) −*x*+1, −*y*+1, −*z*+1; (ii) −*x*+1, −*y*+2, −*z*.

### Hydrogen-bond geometry (Å, º)

**Table:**

*D*—H···*A*	*D*—H	H···*A*	*D*···*A*	*D*—H···*A*
C6—H6···F1^iii^	0.93	2.64	3.562	171

Symmetry code: (iii) −*x*, −*y*+1, −*z*.
